# Evolution of drug resistance in malaria parasites

**DOI:** 10.1186/1475-2875-11-S1-P63

**Published:** 2012-10-15

**Authors:** Mathieu Legros, Sebastian Bonhoeffer

**Affiliations:** 1 ETH Zurich, Institut fur Integrative Biologie, Zurich, Switzerland

## Background

Efforts to relieve the burden caused by malaria rely critically on the availability of drugs targeting *Plasmodium falciparum.* The efficiency of these treatments is however seriously compromised by the appearance and spread of drug resistance. Resistance is observed today to some extent against every available drug, including recent reports of resistance to artemisinin. Resistant strains of *P. falciparum* can spread in affected areas if they fare better than sensitive strains over the entire transmission cycle, including within-human and within-vector infection phases. Both hosts likely represent widely different environments for the parasite, particularly in terms of exposure to drugs and host-specific costs of resistance, which could notably affect the outcome of competition between resistant and sensitive strains and ultimately the evolution of drug resistance.

## Material and methods

To investigate this issue we present a model of malaria transmission combining between-hosts and within-hosts (human and vector) dynamics. The latter incorporates the impact of competition, treatment and immunity in a strain-specific fashion.

## Results

We show how costs of resistance, particularly within-vector costs, affect the selection for resistant strains. We also explore how different drugs, acting on specific parts of the within-human cycle of *P. falciparum*, impact resistant strains (alone or in combination). Finally we also investigate the effect of vector control methods on the prevalence of resistance.

## Conclusions

Weconclude that the evolution of resistance to antimalarial drugs is affected by biological specificities of the parasite across its entire life cycle, including dynamics within both hosts. Theoretical studies in this framework that incorporates parasites, humans and mosquitoes improve our understanding of the emergence and spread of drug resistance, and provide a better picture to investigate sustainable tools in the fight against malaria.

